# Disparities in the Diagnosis and Treatment of Gastric Cancer in Relation to Disabilities

**DOI:** 10.14309/ctg.0000000000000242

**Published:** 2020-10-08

**Authors:** Hyoung Woo Kim, Dong Wook Shin, Kyoung Eun Yeob, In Young Cho, So Young Kim, Seon Mee Park, Jong Heon Park, Jong Hyock Park, Ichiro Kawachi

**Affiliations:** 1 Department of Internal Medicine, Chungbuk National University Hospital, Chungbuk National University, Cheongju, Korea;; 2 Supportive Care Center/Department of Family Medicine, Samsung Medical Center, Sungkyunkwan University School of Medicine, Seoul, Korea;; 3 Department of Digital Health, SAIHST, Sungkyunkwan University, Seoul, Korea;; 4 College of Medicine/Graduate School of Health Science Business Convergence, Chungbuk National University, Cheongju, Korea;; 5 Department of Public Health and Preventive Medicine, Chungbuk National University Hospital, Cheongju, Korea;; 6 Harvard TH Chan School of Public Health, Harvard University, Boston, Massachusetts, USA;; 7 Big Data Steering Department, National Health Insurance Service, Wonju, Korea.

## Abstract

We investigated potential disparities in the diagnosis, treatment, and survival of gastric cancer (GC) patients with and without disabilities.

**METHODS:**

We linked Korean National Disability Registry data with the Korean National Health Insurance database and Korean Central Cancer Registry data. This study included a total of 16,849 people with disabilities and 58,872 age- and sex-matched control subjects in whom GC had been diagnosed.

**RESULTS:**

When compared to GC patients without disabilities, patients with disabilities tended to be diagnosed at a later stage (localized stage 53.7% vs 59.0% or stage unknown 10.7% vs 6.9%), especially those with severe disabilities (*P* < 0.001). This was more evident in patients with mental impairment (localized stage 41.7% and stage unknown 15.2%). In addition, not receiving treatment was more common in patients with disabilities than those without disabilities (29.3% vs 27.2%, *P* < 0.001), and this disparity was more evident in those with severe disabilities (35.4%) and in those with communication (36.9%) and mental (32.3%) impairment. Patients with disabilities were at slightly higher risk of overall mortality as well as GC-specific mortality compared to people without disabilities (adjusted hazard ratio [aHR] = 1.18, 95% confidence interval: 1.14–1.21 and aHR = 1.12, 95% confidence interval: 1.09–1.16, respectively), and these disparities were more pronounced in those with severe disabilities (aHR = 1.62 and 1.51, respectively).

**DISCUSSION:**

Patients with disabilities, especially severe disabilities, were diagnosed with GC at a later stage, received less staging evaluation and treatment, and their overall survival rate was slightly worse compared to those without disabilities.

## INTRODUCTION

As of 2018, gastric cancer (GC) is the sixth most common cancer worldwide (1) and is the third leading cause of death from cancer worldwide (1). Although its global incidence is declining, in 2015, it was still the most common cancer in men, and the fourth most common cancer in women in Korea with an age-standardized incidence rate of 49.3 in men and 20.5 in women per 100,000 (2).

Although the overall prognosis of GC worldwide is still poor (1), it has been improving with earlier detection and advances in treatment. In Korea, GC screening is provided to all people older than 40 years as part of the National Cancer Screening Program (3,4), and the effectiveness of this program was estimated to be a 21% reduction in GC mortality. In addition, improvements have been made in GC treatment, including surgery (5–10) and chemotherapy (11,12). As a result, the age-standardized GC mortality rate declined from 23.8 to 8.9 per 100,000 persons from 1999 to 2015 (2).

People with disabilities represent the largest group of vulnerable populations, and the average prevalence rate in the adult population aged 18 years and older is 15.6% according to the World Health Survey (13). In Korea, even with a narrow definition of disability, the prevalence rate was about 5.4% in 2017 (http://kosis.kr). People with disabilities have physical, communication, psychosocial, and practical barriers to health care access and utilization (14–19). In addition, they often have lower education and income levels (20–23). Thus, they may be diagnosed at a later stage of disease or have an unknown disease status, receive inappropriate or no standard treatment, and have worse survival (24–27).

Therefore, it is important to identify the potential disparities of the cancer diagnosis and treatment between patients with disabilities and those without (24–27). In the United States, a previous study using the Surveillance, Epidemiology, and End Results–Medicare/Social Security Disability Insurance (SSDI) databases examined the potential disparities in the cancer diagnosis and treatment (26). For example, in colorectal cancer, there were no significant diagnostic disparities between people with and without Medicare/SSDI, but people with Medicare/SSDI had higher cancer-specific mortality compared with control (26). However, this study was limited because of restricted assessment of disability depending on Medicare/SSDI status (e.g., people with disabilities who are employed cannot apply for Medicare/SSDI), inclusion of only 5 conditions (mental, neurologic, circulatory, respiratory, and musculoskeletal disorders and exclusion of visual and hearing disabilities), and lack of data on disability severity. Furthermore, only those younger than 65 years were included, limiting the generalizability of results. Probably because of the lower GC incidence in developed countries, there are few studies on the disparities in GC diagnosis and treatment in relation to disabilities.

The Korean is covered by a single-payer, universal health insurance system. The copayment for diagnostic tests and treatment for cancer is capped to 5%, and those in the lowest income bracket are covered by a medical aid program. Furthermore, Korea has a well-established national cancer registry and national disability registration system, which provides an optimal setting for examining cancer care disparities among people with disabilities.

In this study, using the linked administrative database, we investigated potential disparities in the diagnosis, treatment, and survival of GC among people with and without disabilities.

## METHODS

### Study setting and data source

#### Korean National Health System.

The National Health Insurance Service (NHIS) provides obligatory public health insurance for 97% of all Koreans, and the insurance premium is calculated depending on income level. People who are unemployed and have the lowest assets (around 3% of the population) are covered by the Medical Aid program. Healthcare providers deliver medical care and are reimbursed generally through fee-for-service, and submission of healthcare data is required for reimbursement. Therefore, the NHIS has all the data required for reimbursement, which includes demographic data (including age, sex, area of residence, and income level), medical conditions (based on *International Classification of Disease-10* codes), and information on the diagnostic tests and treatment procedures performed, along with a list of the prescriptions. The NHIS has also established a research database (National Health Insurance Research Database), which is available for research purposes. The National Health Insurance Research Database has been used in several epidemiologic and health policy studies (28,29), and further details can be found elsewhere (30,31).

#### Disability registration system in Korea.

A national registration system for people with disabilities was established in 1988, to determine the eligibility for welfare benefits based on disability type and severity. According to legislation, there are 15 categories of disability: brain, facial, visual, auditory, linguistic, heart, respiratory, liver, kidney, ostomy, limb, epilepsy, intellectual, autistic, and mental. A medical specialist evaluated functional losses and clinical impairments that persisted longer than 6 months, and the severity of disability was classified into 6 levels according to government criteria (32). Furthermore, after the initial assessment, they are subject to reassessment and reclassification after 2–3 years, and if their disability seems to be permanent at that time, they are exempt from further reassessment. However, as initial registration requires that functional losses and clinical impairments persist longer than 6 months, most people with disabilities registered in the disability registration system maintain their status. In our study, the types of disabilities were rearranged in to 5 groups: (i) physical (brain impairment and limb disability), (ii) communication (visual, auditory, and linguistic disability), (iii) mental (intellectual, autistic, and mental disability), (iv) internal organ (heart, lung, and renal disability plus ostomy), and (v) other (facial disfigurement and epilepsy). We then dichotomized severity levels into either severe (grades 1–3) or mild (grades 4–6).

#### Cancer registration system in Korea.

The Korean Central Cancer Registry is a government-sponsored, nationwide cancer registry, and includes data on age at diagnosis, sex, date of diagnosis, cancer site, and Surveillance, Epidemiology, and End Results stage.

### Study subjects

First, we linked the Korean NHIS database with national disability registration data and selected 3 control subjects for each subject with any registered disability during 2009–2013 through age- and sex-matching. Second, cancer registration data from Korean Central Cancer Registry were linked to all subjects in the Korean NHIS-disability study data set.

The study population included all subjects who were diagnosed with GC (*International Classification of Disease* code C16) from January 1, 2009, to December 31, 2013 (n = 81,505). Control subjects were assigned an index date, corresponding to the date of the GC diagnosis of their matched GC patients. We excluded patients who (i) were younger than 19 years at diagnosis or index date (n = 1), (ii) had a history of other cancers before the GC diagnosis (n = 4,872), or (iii) had missing data (n = 911).

**Figure 1.**
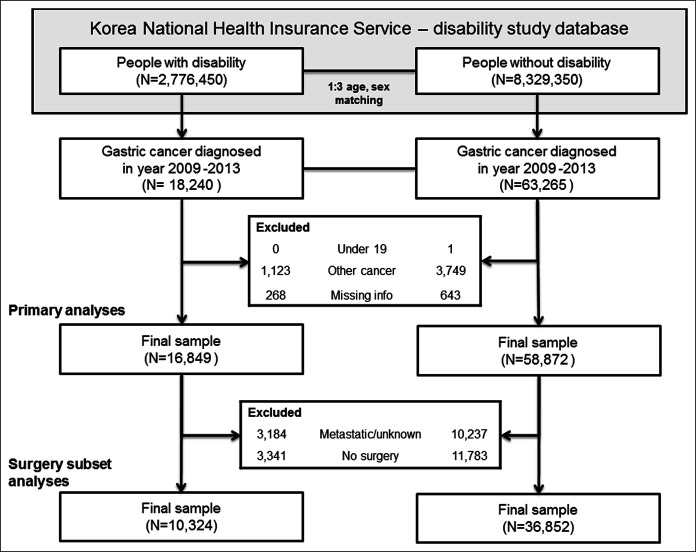
Study participants.

The final sample consisted of 75,721 patients with GC, of which 16,849 had a disability and 58,872 did not. Therefore, the case-to-control ratio was generally well-maintained (case:control = 1:3.49). Finally, we linked our data set to vital statistics provided by the Korean National Statistical Office, which include the date and cause of death (Figure 1).

Institutional Review Board approval was obtained from Chungbuk National University (IRB No. CBNU-201708-BM-501-01).

### Statistical analysis

The summary of statistics includes the presence or absence of disabilities, severity of the disabilities, and the 5 predefined disability categories. Cancer stage and treatment received were also tabulated by disability status, and statistical differences were tested by the χ^2^ test.

Cox regression analysis was used to determine the hazard ratios for the overall and GC-specific mortality for people with disabilities compared with control. Survival was calculated from the GC diagnosis or index date until the date of death, censor date (outmigration or death from other causes for GC-specific mortality), or last follow-up date (December 31, 2015). The multivariable model included age, sex, Charlson comorbidity index (33), income level, residential area, cancer stage, and treatment received. The same analyses were repeated with the surgery subset. All the analyses were performed using SAS statistical software (version 9.4; SAS Institute, Cary, NC). *P* values <0.05 were considered statistically significant.

## RESULTS

### Subject characteristics

GC patients with disabilities were slightly younger than the control subjects (66.0 vs 66.5 years), and those with mental and other impairments were much younger (56.0 and 58.9 years, respectively). They had more comorbidities and higher Charlson comorbidity index scores (1.5 vs 1.1). In addition, they were more likely to be living in rural areas and had lower income levels (Table 1).

**Table 1.**
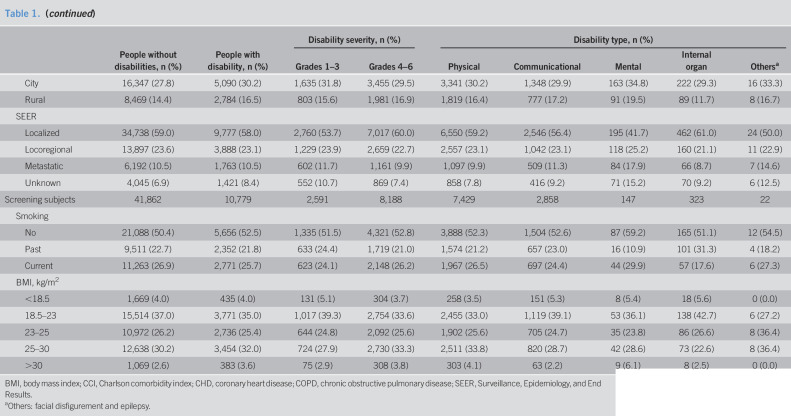
Characteristics of patients with gastric cancer

	People without disabilities, n (%)	People with disability, n (%)	Disability severity, n (%)		Disability type, n (%)				
			Grades 1–3	Grades 4–6	Physical	Communicational	Mental	Internal organ	Others^a^
All subjects	58,872	16,849	5,143	11,706	11,062	4,513	468	758	48
Age, yr									
Mean (SD)	66.5 (10.4)	66.0 (10.5)	65.5 (10.9)	66.2 (10.3)	66.2 (9.6)	69.1 (10.3)	56.0 (11.3)	64.3 (10.2)	58.9 (11.4)
19–40	601 (1.0)	195 (1.1)	90 (1.7)	105 (0.9)	119 (1.1)	33 (0.7)	33 (7.0)	8 (1.1)	2 (4.2)
41–65	21,645 (36.8)	6,465 (38.4)	2,041 (39.7)	4,424 (37.8)	4,532 (41.0)	1,250 (27.7)	320 (68.4)	332 (43.8)	31 (64.6)
66–75	23,391 (39.7)	6,700 (39.8)	1,959 (38.1)	4,741 (40.5)	4,503 (40.7)	1,794 (39.8)	100 (21.4)	291 (38.4)	12 (25.0)
>75	13,235 (22.5)	3,489 (20.7)	1,053 (20.5)	2,436 (20.8)	1,908 (17.2)	1,436 (31.8)	15 (3.2)	127 (16.7)	3 (6.2)
Sex									
Male	43,265 (73.5)	12,397 (73.6)	3,962 (77.0)	8,435 (72.1)	8,043 (72.7)	3,401 (75.4)	314 (67.1)	600 (79.2)	39 (81.3)
Female	15,607 (26.5)	4,452 (26.4)	1,181 (23.0)	3,271 (27.9)	3,019 (27.3)	1,112 (24.6)	154 (32.9)	158 (20.8)	9 (18.7)
CCI									
Mean (SD)	1.1 (1.6)	1.5 (1.9)	1.8 (2.2)	1.5 (1.8)	1.9 (2.0)	1.4 (1.8)	0.9 (1.4)	3.2 (2.6)	1.5 (1.7)
0	30,545 (51.9)	6,977 (41.4)	2,035 (39.6)	4,942 (42.2)	4,531 (41.0)	1,992 (44.1)	279 (59.6)	159 (21.0)	16 (33.3)
1	11,949 (20.3)	3,289 (19.5)	865 (16.8)	2,424 (20.7)	2,187 (19.8)	914 (20.3)	89 (19.0)	84 (11.1)	15 (31.3)
2	7,086 (12.0)	2,363 (14.0)	668 (13.0)	1,695 (14.5)	1,597 (14.4)	613 (13.6)	49 (10.5)	98 (12.9)	6 (12.5)
≥3	9,292 (15.8)	4,220 (25.1)	1,575 (30.6)	2,645 (22.6)	2,747 (24.8)	994 (22.0)	51 (10.9)	417 (55.0)	11 (22.9
Comorbidity									
Diabetes mellitus	9,399 (16.0)	3,318 (19.7)	1,053 (20.5)	2,265 (19.4)	2,146 (19.4)	878 (19.5)	40 (8.6)	249 (32.9)	5 (10.4)
Hypertension	23,258 (39.5)	7,622 (45.2)	2,411 (46.9)	5,211 (44.5)	5,096 (46.1)	1,960 (43.4)	92 (19.7)	460 (60.7)	14 (29.2)
CHD	6,813 (11.6)	2,472 (14.7)	878 (17.1)	1,594 (13.6)	1,547 (14.0)	625 (13.9)	26 (5.6)	269 (35.5)	5 (10.4)
Stroke	3,332 (5.7)	2,083 (12.4)	931 (18.1)	1,152 (9.8)	1,565 (14.2)	413 (9.2)	23 (4.9)	77 (10.2)	5 (10.4)
COPD	7,136 (12.2)	2,553 (15.2)	852 (16.6)	1,701 (14.5)	1,577 (14.3)	707 (15.7)	45 (9.6)	218 (28.8)	6 (12.5)
Income									
Medicare	2,569 (4.4)	1,986 (11.8)	1,054 (20.5)	932 (7.9)	1,100 (9.9)	478 (10.6)	280 (59.8)	110 (14.5)	18 (37.5)
Lowest quartile	13,141 (22.3)	3,852 (22.9)	1,034 (20.1)	2,818 (24.1)	2,564 (23.2)	1,041 (23.1)	71 (15.2)	165 (21.8)	11 (22.9)
Second quartile	11,508 (19.6)	3,156 (18.7)	852 (16.6)	2,304 (19.7)	2,192 (19.8)	795 (17.6)	36 (7.7)	126 (16.6)	7 (14.6)
Third quartile	14,200 (24.1)	3,744 (22.2)	1,055 (20.5)	2,689 (23.0)	2,536 (22.9)	980 (21.7)	41 (8.8)	183 (24.1)	4 (8.3)
Highest quartile	17,454 (29.6)	4,111 (24.4)	1,148 (22.3)	2,963 (25.3)	2,670 (24.2)	1,219 (27.0)	40 (8.5)	174 (23.0)	8 (16.7)
Residence									
Metropolitan	34,056 (57.8)	8,975 (53.3)	2,705 (52.6)	6,270 (53.6)	5,902 (53.4)	2,388 (52.9)	214 (45.7)	447 (59.0)	24 (50.0)
City	16,347 (27.8)	5,090 (30.2)	1,635 (31.8)	3,455 (29.5)	3,341 (30.2)	1,348 (29.9)	163 (34.8)	222 (29.3)	16 (33.3)
Rural	8,469 (14.4)	2,784 (16.5)	803 (15.6)	1,981 (16.9)	1,819 (16.4)	777 (17.2)	91 (19.5)	89 (11.7)	8 (16.7)
SEER									
Localized	34,738 (59.0)	9,777 (58.0)	2,760 (53.7)	7,017 (60.0)	6,550 (59.2)	2,546 (56.4)	195 (41.7)	462 (61.0)	24 (50.0)
Locoregional	13,897 (23.6)	3,888 (23.1)	1,229 (23.9)	2,659 (22.7)	2,557 (23.1)	1,042 (23.1)	118 (25.2)	160 (21.1)	11 (22.9)
Metastatic	6,192 (10.5)	1,763 (10.5)	602 (11.7)	1,161 (9.9)	1,097 (9.9)	509 (11.3)	84 (17.9)	66 (8.7)	7 (14.6)
Unknown	4,045 (6.9)	1,421 (8.4)	552 (10.7)	869 (7.4)	858 (7.8)	416 (9.2)	71 (15.2)	70 (9.2)	6 (12.5)
Screening subjects	41,862	10,779	2,591	8,188	7,429	2,858	147	323	22
Smoking									
No	21,088 (50.4)	5,656 (52.5)	1,335 (51.5)	4,321 (52.8)	3,888 (52.3)	1,504 (52.6)	87 (59.2)	165 (51.1)	12 (54.5)
Past	9,511 (22.7)	2,352 (21.8)	633 (24.4)	1,719 (21.0)	1,574 (21.2)	657 (23.0)	16 (10.9)	101 (31.3)	4 (18.2)
Current	11,263 (26.9)	2,771 (25.7)	623 (24.1)	2,148 (26.2)	1,967 (26.5)	697 (24.4)	44 (29.9)	57 (17.6)	6 (27.3)
BMI, kg/m^2^									
<18.5	1,669 (4.0)	435 (4.0)	131 (5.1)	304 (3.7)	258 (3.5)	151 (5.3)	8 (5.4)	18 (5.6)	0 (0.0)
18.5–23	15,514 (37.0)	3,771 (35.0)	1,017 (39.3)	2,754 (33.6)	2,455 (33.0)	1,119 (39.1)	53 (36.1)	138 (42.7)	6 (27.2)
23–25	10,972 (26.2)	2,736 (25.4)	644 (24.8)	2,092 (25.6)	1,902 (25.6)	705 (24.7)	35 (23.8)	86 (26.6)	8 (36.4)
25–30	12,638 (30.2)	3,454 (32.0)	724 (27.9)	2,730 (33.3)	2,511 (33.8)	820 (28.7)	42 (28.6)	73 (22.6)	8 (36.4)
>30	1,069 (2.6)	383 (3.6)	75 (2.9)	308 (3.8)	303 (4.1)	63 (2.2)	9 (6.1)	8 (2.5)	0 (0.0)

BMI, body mass index; CCI, Charlson comorbidity index; CHD, coronary heart disease; COPD, chronic obstructive pulmonary disease; SEER, Surveillance, Epidemiology, and End Results.

a Others: facial disfigurement and epilepsy.

### Disease status by disability characteristics

In general, people with disabilities had similar stage distributions compared to people without disabilities, except that they were slightly more likely to have an unknown disease status (8.4% vs 6.9%). However, the localized stage was lower in people with severe disabilities (53.7% for grades 1–3 and 46.8% for grade 1), whereas the unknown stage was more common (10.7% for grades 1–3 and 15.1% for grade 1). Among disability types, people with mental impairment tended to be diagnosed at a later disease status (localized stage 41.7%) and were more likely to have an unknown stage (15.2%) (Table 2).

**Table 2.**
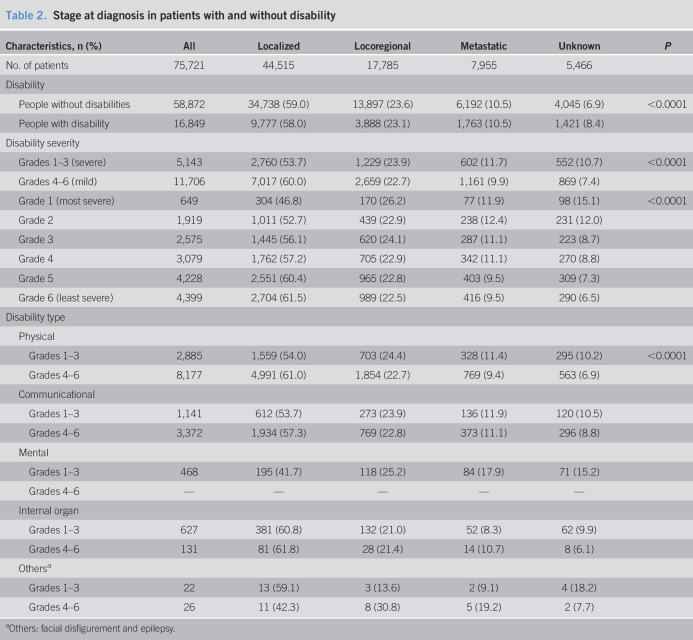
Stage at diagnosis in patients with and without disability

Characteristics, n (%)	All	Localized	Locoregional	Metastatic	Unknown	*P*
No. of patients	75,721	44,515	17,785	7,955	5,466	
Disability						
People without disabilities	58,872	34,738 (59.0)	13,897 (23.6)	6,192 (10.5)	4,045 (6.9)	<0.0001
People with disability	16,849	9,777 (58.0)	3,888 (23.1)	1,763 (10.5)	1,421 (8.4)	
Disability severity						
Grades 1–3 (severe)	5,143	2,760 (53.7)	1,229 (23.9)	602 (11.7)	552 (10.7)	<0.0001
Grades 4–6 (mild)	11,706	7,017 (60.0)	2,659 (22.7)	1,161 (9.9)	869 (7.4)	
Grade 1 (most severe)	649	304 (46.8)	170 (26.2)	77 (11.9)	98 (15.1)	<0.0001
Grade 2	1,919	1,011 (52.7)	439 (22.9)	238 (12.4)	231 (12.0)	
Grade 3	2,575	1,445 (56.1)	620 (24.1)	287 (11.1)	223 (8.7)	
Grade 4	3,079	1,762 (57.2)	705 (22.9)	342 (11.1)	270 (8.8)	
Grade 5	4,228	2,551 (60.4)	965 (22.8)	403 (9.5)	309 (7.3)	
Grade 6 (least severe)	4,399	2,704 (61.5)	989 (22.5)	416 (9.5)	290 (6.5)	
Disability type						
Physical						
Grades 1–3	2,885	1,559 (54.0)	703 (24.4)	328 (11.4)	295 (10.2)	<0.0001
Grades 4–6	8,177	4,991 (61.0)	1,854 (22.7)	769 (9.4)	563 (6.9)	
Communicational						
Grades 1–3	1,141	612 (53.7)	273 (23.9)	136 (11.9)	120 (10.5)	
Grades 4–6	3,372	1,934 (57.3)	769 (22.8)	373 (11.1)	296 (8.8)	
Mental						
Grades 1–3	468	195 (41.7)	118 (25.2)	84 (17.9)	71 (15.2)	
Grades 4–6	—	—	—	—	—	
Internal organ						
Grades 1–3	627	381 (60.8)	132 (21.0)	52 (8.3)	62 (9.9)	
Grades 4–6	131	81 (61.8)	28 (21.4)	14 (10.7)	8 (6.1)	
Others^a^						
Grades 1–3	22	13 (59.1)	3 (13.6)	2 (9.1)	4 (18.2)	
Grades 4–6	26	11 (42.3)	8 (30.8)	5 (19.2)	2 (7.7)	

a Others: facial disfigurement and epilepsy.

### Treatment patterns by disability characteristics

People with disabilities were less likely to undergo surgery (65.1% vs 66.2%), perioperative chemotherapy (8.8% vs 9.5%), and palliative chemotherapy (5.6% vs 6.6%) and also tended to have no cancer treatment at all compare with the control subjects (29.3% vs 27.2%). This trend was more prominent in patients with severe disabilities than in those with mild disabilities (50.4% vs 58.3% in surgery alone, 8.3% vs 9.0% in surgery plus chemotherapy, 5.6% vs 5.6% in palliative chemotherapy, and 35.4% vs 26.6% in no treatment, respectively). By disability type, not receiving treatment was more common for communication impairment (36.9% in severe disability and 31.4% in mild disability) and mental impairment (32.3%) (Table 3 and see Supplementary Table 1, Supplementary Digital Content 1, http://links.lww.com/CTG/A395).

**Table 3.**
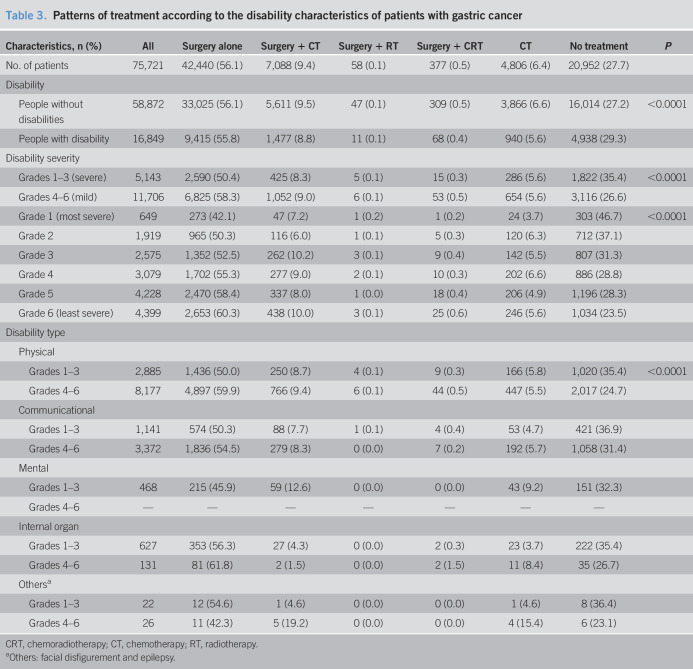
Patterns of treatment according to the disability characteristics of patients with gastric cancer

Characteristics, n (%)	All	Surgery alone	Surgery + CT	Surgery + RT	Surgery + CRT	CT	No treatment	*P*
No. of patients	75,721	42,440 (56.1)	7,088 (9.4)	58 (0.1)	377 (0.5)	4,806 (6.4)	20,952 (27.7)	
Disability								
People without disabilities	58,872	33,025 (56.1)	5,611 (9.5)	47 (0.1)	309 (0.5)	3,866 (6.6)	16,014 (27.2)	<0.0001
People with disability	16,849	9,415 (55.8)	1,477 (8.8)	11 (0.1)	68 (0.4)	940 (5.6)	4,938 (29.3)	
Disability severity								
Grades 1–3 (severe)	5,143	2,590 (50.4)	425 (8.3)	5 (0.1)	15 (0.3)	286 (5.6)	1,822 (35.4)	<0.0001
Grades 4–6 (mild)	11,706	6,825 (58.3)	1,052 (9.0)	6 (0.1)	53 (0.5)	654 (5.6)	3,116 (26.6)	
Grade 1 (most severe)	649	273 (42.1)	47 (7.2)	1 (0.2)	1 (0.2)	24 (3.7)	303 (46.7)	<0.0001
Grade 2	1,919	965 (50.3)	116 (6.0)	1 (0.1)	5 (0.3)	120 (6.3)	712 (37.1)	
Grade 3	2,575	1,352 (52.5)	262 (10.2)	3 (0.1)	9 (0.4)	142 (5.5)	807 (31.3)	
Grade 4	3,079	1,702 (55.3)	277 (9.0)	2 (0.1)	10 (0.3)	202 (6.6)	886 (28.8)	
Grade 5	4,228	2,470 (58.4)	337 (8.0)	1 (0.0)	18 (0.4)	206 (4.9)	1,196 (28.3)	
Grade 6 (least severe)	4,399	2,653 (60.3)	438 (10.0)	3 (0.1)	25 (0.6)	246 (5.6)	1,034 (23.5)	
Disability type								
Physical								
Grades 1–3	2,885	1,436 (50.0)	250 (8.7)	4 (0.1)	9 (0.3)	166 (5.8)	1,020 (35.4)	<0.0001
Grades 4–6	8,177	4,897 (59.9)	766 (9.4)	6 (0.1)	44 (0.5)	447 (5.5)	2,017 (24.7)	
Communicational								
Grades 1–3	1,141	574 (50.3)	88 (7.7)	1 (0.1)	4 (0.4)	53 (4.7)	421 (36.9)	
Grades 4–6	3,372	1,836 (54.5)	279 (8.3)	0 (0.0)	7 (0.2)	192 (5.7)	1,058 (31.4)	
Mental								
Grades 1–3	468	215 (45.9)	59 (12.6)	0 (0.0)	0 (0.0)	43 (9.2)	151 (32.3)	
Grades 4–6	—	—	—	—	—	—	—	
Internal organ								
Grades 1–3	627	353 (56.3)	27 (4.3)	0 (0.0)	2 (0.3)	23 (3.7)	222 (35.4)	
Grades 4–6	131	81 (61.8)	2 (1.5)	0 (0.0)	2 (1.5)	11 (8.4)	35 (26.7)	
Others^a^								
Grades 1–3	22	12 (54.6)	1 (4.6)	0 (0.0)	0 (0.0)	1 (4.6)	8 (36.4)	
Grades 4–6	26	11 (42.3)	5 (19.2)	0 (0.0)	0 (0.0)	4 (15.4)	6 (23.1)	

CRT, chemoradiotherapy; CT, chemotherapy; RT, radiotherapy.

a Others: facial disfigurement and epilepsy.

### Survival in all patients with GC

In total, 37.1% (28,071 of 75,721) of patients with GC died during an average follow-up of 3.4 years. People with disabilities had a slightly higher risk of mortality than those without disabilities (adjusted hazard ratio [aHR] = 1.18, 95% confidence interval [CI]: 1.14–1.21). Moreover, this difference was more prominent in the severe disability group (aHR = 1.62, 95% CI: 1.56–1.69), whereas overall mortality risk in the mild disability group was marginally higher than those in the control subjects (aHR = 1.05, 95% CI: 1.01–1.08). Likewise, by disability type, the risk of overall mortality was consistently higher in the severe disability group with physical impairment (aHR = 1.48, 95% CI: 1.40–1.56), communication impairment (aHR = 1.31, 95% CI: 1.21–1.43), mental impairment (aHR = 2.06, 95% CI: 1.82–2.34), internal organ impairment (aHR = 1.89, 95% CI: 1.70–2.10), and other impairment (aHR = 1.96, 95% CI: 1.11–3.46).

GC itself accounted for 76.9% of all deaths (21,595 of 28,071). GC-specific mortality risk showed a similar pattern to the overall mortality risk except for lower HR in people with internal organ impairment (Table 4).

**Table 4.**
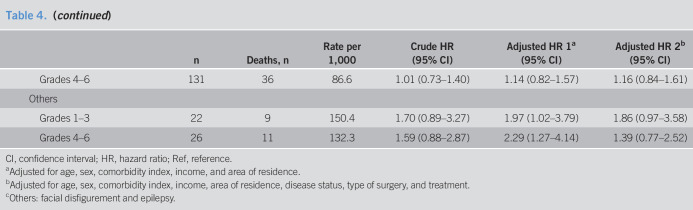
Overall and cancer-specific mortality risk of patients with gastric cancer of all stages

	n	Deaths, n	Rate per 1,000	Crude HR (95% CI)	Adjusted HR 1^a^ (95% CI)	Adjusted HR 2^b^ (95% CI)
Overall mortality						
Disability						
People without disabilities	58,872	21,199	104.3	Ref	Ref	Ref
People with disability	16,849	6,872	125.2	1.19 (1.16–1.22)	1.17 (1.14–1.21)	1.18 (1.14–1.21)
Disability severity						
Grades 1–3	5,143	2,657	177.4	1.65 (1.58–1.72)	1.63 (1.56–1.70)	1.62 (1.56–1.69)
Grades 4–6	11,706	4,215	105.6	1.01 (0.98–1.04)	1.00 (0.97–1.04)	1.05 (1.01–1.08)
Disability type						
Physical						
Grades 1–3	2,885	1,458	172.0	1.60 (1.52–1.69)	1.65 (1.56–1.74)	1.48 (1.40–1.56)
Grades 4–6	8,177	2,696	94.3	0.91 (0.87–0.94)	0.95 (0.92–0.99)	1.00 (0.96–1.04)
Communicational						
Grades 1–3	1,141	568	167.2	1.56 (1.44–1.70)	1.28 (1.18–1.39)	1.31 (1.21–1.43)
Grades 4–6	3,372	1,455	134.5	1.27 (1.20–1.34)	1.10 (1.04–1.16)	1.11 (1.05–1.17)
Mental						
Grades 1–3	468	253	201.6	1.86 (1.65–2.11)	2.53 (2.23–2.88)	2.06 (1.82–2.34)
Grades 4–6	—	—	—	—	—	—
Internal organ						
Grades 1–3	627	366	204.0	1.86 (1.68–2.06)	1.93 (1.74–2.14)	1.89 (1.70–2.10)
Grades 4–6	131	52	125.2	1.16 (0.89–1.53)	1.23 (0.94–1.61)	1.26 (0.96–1.65)
Others^c^						
Grades 1–3	22	12	200.6	1.84 (1.05–3.24)	1.99 (1.13–3.51)	1.96 (1.11–3.46)
Grades 4–6	26	12	144.4	1.37 (0.78–2.41)	2.02 (1.14–3.55)	1.27 (0.72–2.23)
Gastric cancer–specific mortality						
Disability						
People without disabilities	58,872	16,541	81.3	Ref	Ref	Ref
People with disability	16,849	5,054	92	1.11 (1.07–1.14)	1.12 (1.08–1.15)	1.12 (1.09–1.16)
Disability severity						
Grades 1–3	5,143	1,907	127.3	1.49 (1.42–1.56)	1.52 (1.45–1.59)	1.51 (1.44–1.58)
Grades 4–6	11,706	3,147	78.8	0.96 (0.92–1.00)	0.97 (0.93–1.01)	1.02 (0.98–1.06)
Disability type						
Physical						
Grades 1–3	2,885	1,074	126.7	1.49 (1.40–1.58)	1.57 (1.48–1.68)	1.37 (1.29–1.46)
Grades 4–6	8,177	1,999	69.9	0.86 (0.82–0.90)	0.91 (0.87–0.96)	0.97 (0.92–1.01)
Communicational						
Grades 1–3	1,141	446	131.3	1.55 (1.42–1.71)	1.31 (1.19–1.44)	1.33 (1.21–1.46)
Grades 4–6	3,372	1,101	101.7	1.22 (1.15–1.29)	1.08 (1.01–1.14)	1.08 (1.02–1.15)
Mental						
Grades 1–3	468	217	172.8	2.02 (1.77–2.31)	2.67 (2.32–3.06)	2.13 (1.85–2.44)
Grades 4–6	—	—	—	—	—	—
Internal organ						
Grades 1–3	627	161	89.7	1.02 (0.88–1.20)	1.14 (0.98–1.33)	1.12 (0.96–1.31)
Grades 4–6	131	36	86.6	1.01 (0.73–1.40)	1.14 (0.82–1.57)	1.16 (0.84–1.61)
Others						
Grades 1–3	22	9	150.4	1.70 (0.89–3.27)	1.97 (1.02–3.79)	1.86 (0.97–3.58)
Grades 4–6	26	11	132.3	1.59 (0.88–2.87)	2.29 (1.27–4.14)	1.39 (0.77–2.52)

CI, confidence interval; HR, hazard ratio; Ref, reference.

a Adjusted for age, sex, comorbidity index, income, and area of residence.

b Adjusted for age, sex, comorbidity index, income, area of residence, disease status, type of surgery, and treatment.

c Others: facial disfigurement and epilepsy.

### Treatment patterns and survival in patients with resected GC

Among patients who underwent surgical treatment, people with disabilities received adjuvant therapy at a similar rate to people without disabilities (13.0% vs 13.9%), including those with severe disabilities (13.5%). People with internal organ impairment were less likely to receive adjuvant therapy (6.1%) (see Supplementary Table 2, Supplementary Digital Content 1, http://links.lww.com/CTG/A395).

People with disabilities had a higher risk of overall mortality than those without disabilities (aHR = 1.21, 95% CI: 1.16–1.27). This difference was more marked in the severe disability group (aHR = 1.69, 95% CI: 1.57–1.81), but was not significant in the mild disability group (aHR = 1.05, 95% CI: 0.99–1.11). In the severe disability group, the risk was significantly higher across all disability types (aHR = 1.64 in physical impairment, aHR = 1.24 in communication impairment, aHR = 1.88 in mental impairment, aHR = 2.83 in internal organ impairment, and aHR = 3.53 in other impairments). The above-mentioned estimates were generally consistent with those for GC-specific mortality (Table 5).

**Table 5.**
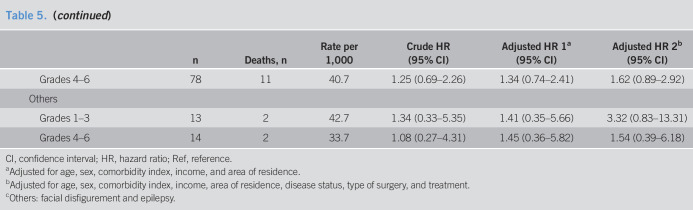
Risk of overall and cancer-specific mortality for patients with resected gastric cancer

	n	Deaths, n	Rate per 1,000	Crude HR (95% CI)	Adjusted HR 1^a^ (95% CI)	Adjusted HR 2^b^ (95% CI)
Overall mortality						
Disability						
People without disabilities	36,852	7,243	50.0	Ref	Ref	Ref
People with disability	10,324	2,412	61.2	1.23 (1.18–1.29)	1.18 (1.13–1.24)	1.21 (1.16–1.27)
Disability severity						
Grades 1–3	2,831	894	86.7	1.74 (1.63–1.87)	1.64 (1.53–1.76)	1.69 (1.57–1.81)
Grades 4–6	7,493	1,518	52.2	1.05 (0.99–1.11)	1.02 (0.97–1.08)	1.05 (0.99–1.11)
Disability type						
Physical						
Grades 1–3	1,600	482	82.1	1.65 (1.51–1.81)	1.62 (1.48–1.78)	1.64 (1.49–1.80)
Grades 4–6	5,397	1,011	48.0	0.97 (0.90–1.03)	0.98 (0.92–1.05)	1.01 (0.95–1.08)
Communicational						
Grades 1–3	620	173	74.1	1.48 (1.27–1.72)	1.19 (1.02–1.38)	1.24 (1.07–1.45)
Grades 4–6	2,004	485	63.1	1.27 (1.16–1.39)	1.11 (1.01–1.21)	1.12 (1.02–1.23)
Mental						
Grades 1–3	237	71	82.4	1.67 (1.32–2.11)	2.05 (1.61–2.61)	1.88 (1.48–2.39)
Grades 4–6	—	—	—	—	—	—
Internal organ						
Grades 1–3	361	163	135.7	2.74 (2.34–3.20)	2.59 (2.21–3.02)	2.83 (2.42–3.31)
Grades 4–6	78	19	70.4	1.46 (0.93–2.29)	1.41 (0.90–2.22)	1.60 (1.02–2.50)
Others^c^						
Grades 1–3	13	5	106.6	2.21 (0.92–5.32)	2.24 (0.93–5.39)	3.53 (1.47–8.50)
Grades 4–6	14	3	50.6	1.01 (0.33–3.14)	1.46 (0.47–4.53)	1.44 (0.46–4.46)
Gastric cancer–specific mortality						
Disability						
People without disabilities	36,852	4,607	31.8	Ref	Ref	Ref
People with disability	10,324	1,394	35.4	1.11 (1.05–1.18)	1.09 (1.03–1.16)	1.13 (1.06–1.20)
Disability severity						
Grades 1–3	2,831	475	46.1	1.44 (1.31–1.59)	1.41 (1.28–1.56)	1.47 (1.33–1.62)
Grades 4–6	7,493	919	31.6	0.99 (0.92–1.06)	0.99 (0.92–1.06)	1.02 (0.95–1.09)
Disability type						
Physical						
Grades 1–3	1,600	271	46.2	1.45 (1.28–1.64)	1.47 (1.30–1.66)	1.48 (1.31–1.68)
Grades 4–6	5,397	606	28.8	0.90 (0.83–0.98)	0.93 (0.85–1.01)	0.97 (0.89–1.05)
Communicational						
Grades 1–3	620	98	42.0	1.32 (1.08–1.61)	1.12 (0.92–1.37)	1.17 (0.96–1.43)
Grades 4–6	2,004	300	39.0	1.22 (1.09–1.38)	1.11 (0.99–1.25)	1.12 (0.99–1.25)
Mental						
Grades 1–3	237	50	58.0	1.82 (1.38–2.41)	2.07 (1.56–2.76)	1.83 (1.37–2.43)
Grades 4–6	—	—	—	—	—	—
Internal organ						
Grades 1–3	361	54	45.0	1.39 (1.07–1.82)	1.45 (1.11–1.90)	1.67 (1.28–2.19)
Grades 4–6	78	11	40.7	1.25 (0.69–2.26)	1.34 (0.74–2.41)	1.62 (0.89–2.92)
Others						
Grades 1–3	13	2	42.7	1.34 (0.33–5.35)	1.41 (0.35–5.66)	3.32 (0.83–13.31)
Grades 4–6	14	2	33.7	1.08 (0.27–4.31)	1.45 (0.36–5.82)	1.54 (0.39–6.18)

CI, confidence interval; HR, hazard ratio; Ref, reference.

a Adjusted for age, sex, comorbidity index, income, and area of residence.

b Adjusted for age, sex, comorbidity index, income, area of residence, disease status, type of surgery, and treatment.

c Others: facial disfigurement and epilepsy.

## DISCUSSION

This is the first study to undertake a comprehensive investigation into the potential disparity between GC care and disabilities. Our study showed that GC patients with disabilities, especially those with severe disability and mental impairment, are likely to be diagnosed at a later stage, receive less adequate treatment, and have worse clinical prognosis compared with patients free of disabilities. The particular strengths of our study include the large and representative samples covering the whole nation, assessment of a comprehensive range of disabling conditions, and objective assessment of the disability type and severity.

We found that people with severe disabilities and mental impairments tend to be diagnosed at a later stage. They face practical barriers to primary care utilization because of physical access and communication barriers (34), and they have been reported to undergoing cancer screening at lower rates compared to people without disabilities because of a lack of awareness of care recommendations and difficulty understanding the importance of screening (15–19). For example, significant disparities were found in GC, colorectal cancer, and cervical cancer screening, especially in patients with severe brain-related or mental disabilities in Korea and the United States (15–19). Therefore, we suspect that lower cancer screening rates as well as lower rates of primary care utilization might be associated with later diagnosis in people with severe disabilities and mental impairments.

We found that the stage of disease was more likely to be marked “unknown” among people with disabilities. Like a previous study on disparities in lung cancer (24), this trend was more evident in the severe disability group and among those with mental impairments. GC staging may be “unknown” because patients did not receive proper staging tests to establish an appropriate treatment plan, which means that they probably gave up subsequent treatment (35). However, disability itself is not a contraindication for receiving cancer treatment. These results may reflect the ableism inherent in patients, family members, or healthcare providers, which is an attitude that devalues or sets limitations on the capabilities of people with disabilities.

Our study showed that people with disabilities were more likely to receive no cancer treatment and were less likely to undergo surgery or chemotherapy, especially when their disability is severe. Our findings imply that people with disabilities may be discouraged from receiving cancer treatment by their healthcare providers or family members, who may undervalue the benefits of treatment and overemphasize complications in patients with disabilities (24,25,27). Furthermore, receiving less treatment was more evident in people with communication and mental impairments. These patients often have difficulty in communicating with healthcare providers and limited access to oncological information (23,24,36,37). In addition, decision-making capability may limit the patients with mental impairment of treating their own oncological problems (23,24,36). Therefore, it is necessary to develop medically specialized communication and decision aids to fit the needs and to optimize cancer treatment in patients with disabilities (38).

After adjustment for the patients' characteristics, disease status, and treatment, patients with disabilities had a higher overall and GC-specific mortality. As death from GC comprises most of the deaths (5,054/6,872, 73.5%) in our population, it generally reflects the excess risk of GC death, but other causes can also account for excess mortality, at least partly. Excess GC-specific death in patients with disabilities could be due to the real-life practice of offering less intensive treatment or poor compliance with treatment. Communication or cognitive abilities are important in making adequate decisions and in adherence to cancer treatment (23,24,36,37) and would explain the higher mortality in patients with communication or mental impairment. In addition, death from other causes would be higher in patients with disabilities because they have more comorbidities and poor socioeconomic conditions (24–27). This was evidenced by a higher risk of overall mortality (aHR = 1.89) compared with GC-specific mortality (aHR = 1.12). Potential strategies to reduce prognostic disparities may include the optimization of cancer treatment by overcoming the ableism of family members or healthcare providers and providing socioeconomic aid for people with GC and disabilities (24–27).

Similar to above, overall and GC-specific mortalities were slightly higher in people with resected GC and disabilities (aHR = 1.21) and more pronounced in those with severe disabilities (aHR = 1.68). Among patients with different disability types, people with mental impairment had higher GC-specific mortality (aHR = 1.83), although more of them received adjuvant chemotherapy, suggesting the possibility of less intensive surgery, incompletion of scheduled chemotherapy, or poorly controlled GC care, probably because of communication barriers with healthcare providers (39–42). People with severe physical impairment also had higher GC-specific mortality (aHR = 1.48), suggesting the possibility of less intensive oncologic treatment, probably because of higher risk of recurrent stroke or postoperative ileus (43,44). Therefore, to overcome the disparities in treatment outcomes, appropriate selection of the recipients of surgery and/or intensive adjuvant therapy and rigorous postoperative care may be required.

This study was a retrospective cohort design and had several limitations. First, unknown disease status accounted for 7.2% of all patients, and also, 27.7% of all patients did not receive oncological treatment. However, we do not know why these patients did not receive staging work-up or oncological treatment (e.g. patient or family refusal, clinical judgment by healthcare providers, or economic problems). Second, we did not have detailed clinical information on type of surgical procedure (e.g., laparoscopic or open resection), postoperative morbidity and mortality, pathologic results, adequacy of adjuvant or palliative treatment (e.g., chemotherapy dose, chemotherapy cycles, or number of radiotherapy), medication history such as proton pump inhibitor use, aspirin, or statin use, *Helicobacter pylori* status, or adherence to supportive care, which may have been helpful for interpreting disparities in treatment outcomes.

In conclusion, patients with GC and disabilities, especially severe disabilities, are diagnosed at a later stage, received less staging evaluation and treatment, and their overall survival rate was slightly worse than those without disabilities. This was more evident in people with mental impairment, although they generally do not have physical reasons to receive less screening, diagnostic work-up, and treatment. Although some degree of disparity might be due to rational clinical decisions, a large portion of the disparity seems to be unjustifiable. Efforts should be made to decrease the diagnostic, therapeutic, and prognostic disparities related to disabilities in GC care.

## CONFLICTS OF INTEREST

**Guarantor of the article:** Jong Hyock Park, MD, MPH, PhD.

**Specific author contributions:** Hyoung Woo Kim, MD, and Dong Wook Shin, MD, DrPH, MBA, contributed equally to this work as first author. D.W.S. and J.H.P.: study design. K.E.Y., S.Y.K., and J.H.P.: investigation. S.Y.K. and J.H.P.: resources. H.W.K., D.W.S., J.H.P., and J.H.P.: analysis and interpretation. H.W.K. and D.W.S.: writing of manuscript. I.Y.C., S.M.P., J.H.P., and I.K.: review and editing. D.W.S., J.H.P., and I.K.: supervision.

**Financial support:** This work was supported by the R&D grant (No. 2016007) on rehabilitation by Korea National Rehabilitation Center Research Institute, Ministry of Health & Welfare, and the National Research Foundation of Korea (NRF) grant funded by the Ministry of Education (No. 2019R1H1A2080180, 2019R1A2C1087507).

**Potential competing interests:** None to report.

**Study Highlights**

**WHAT IS KNOWN**

✓ People with disabilities have physical, communication, psychosocial, and practical barriers.
✓ They represent a potentially vulnerable group with respect to access to healthcare system.

**WHAT IS NEW HERE**

✓ People with disabilities were diagnosed with gastric cancer at a later stage.
✓ They received less cancer staging evaluation and treatment and had a slightly higher mortality.

**TRANSLATIONAL IMPACT**

✓ Diagnostic, therapeutic, and prognostic disparities were pronounced in people with severe disability and mental impairment.

## Supplementary Material

SUPPLEMENTARY MATERIAL
