# Supplementary material for: Disparities in the Diagnosis and Treatment of Gastric Cancer in Relation to Disabilities
Source: Clin Transl Gastroenterol. 2020 Oct 8;11(10):e00242. doi: 10.14309/ctg.0000000000000242 (PMC7544185; doi:10.14309/ctg.0000000000000242)
Supplement: SUPPLEMENTARY MATERIAL [file ct9-11-e00242-s001.docx]

**Supplementary Table 1. Patterns of treatment according to the types of disability in patients with gastric cancer.**

| **Characteristics, no. (%)** | **All** | **Surgery alone** | **Surgery + CT** | **Surgery + RT** | **Surgery + CRT** | **CT** | **No treatment** | ***P* value** |
| --- | --- | --- | --- | --- | --- | --- | --- | --- |
| **No. of patients** | 75,721 | 42440 (56.1) | 7088 (9.4) | 58 (0.1) | 377 (0.5) | 4806 (6.4) | 20952 (27.7) |  |
| **Disability** |  |  |  |  |  |  |  |  |
| People without disabilities | 58,872 | 33025 (56.1) | 5611 (9.5) | 47 (0.1) | 309 (0.5) | 3866 (6.6) | 16014 (27.2) | <.0001 |
| People with disability | 16,849 | 9415 (55.9) | 1477 (8.8) | 11 (0.1) | 68 (0.4) | 940 (5.6) | 4938 (29.3) |  |
| **Disability type** |  |  |  |  |  |  |  |  |
| Limb disability |  |  |  |  |  |  |  |  |
| Grade 1–3 | 1,756 | 976 (55.6) | 181 (10.3) | 3 (0.2) | 8 (0.5) | 111 (6.3) | 477 (27.2) | <.0001 |
| Grade 4–6 | 7,556 | 4571 (60.5) | 714 (9.5) | 6 (0.1) | 41 (0.5) | 407 (5.4) | 1817 (24.1) |  |
| Brain disability |  |  |  |  |  |  |  |  |
| Grade 1–3 | 1,129 | 460 (40.7) | 69 (6.1) | 1 (0.1) | 1 (0.1) | 55 (4.9) | 543 (48.1) |  |
| Grade 4–6 | 621 | 326 (52.5) | 52 (8.4) | 0 (0.0) | 3 (0.5) | 40 (6.4) | 200 (32.2) |  |
| Visual disability |  |  |  |  |  |  |  |  |
| Grade 1–3 | 330 | 162 (49.1) | 24 (7.3) | 0 (0.0) | 1 (0.3) | 13 (3.9) | 130 (39.4) |  |
| Grade 4–6 | 1,596 | 928 (58.2) | 146 (9.2) | 0 (0.0) | 3 (0.2) | 102 (6.4) | 417 (26.1) |  |
| Auditory disability |  |  |  |  |  |  |  |  |
| Grade 1–3 | 757 | 382 (50.5) | 60 (7.9) | 1 (0.1) | 2 (0.3) | 37 (4.9) | 275 (36.3) |  |
| Grade 4–6 | 1,739 | 892 (51.3) | 128 (7.4) | 0 (0.0) | 4 (0.2) | 86 (5.0) | 629 (36.2) |  |
| Linguistic disability |  |  |  |  |  |  |  |  |
| Grade 1–3 | 54 | 30 (55.6) | 4 (7.4) | 0 (0.0) | 1 (1.9) | 3 (5.6) | 16 (29.6) |  |
| Grade 4–6 | 37 | 16 (43.2) | 5 (13.5) | 0 (0.0) | 0 (0.0) | 4 (10.8) | 12 (32.4) |  |
| Facial disfigurement |  |  |  |  |  |  |  |  |
| Grade 1–3 | 7 | 3 (42.9) | 0 (0.0) | 0 (0.0) | 0 (0.0) | 1 (14.3) | 3 (42.9) |  |
| Grade 4–6 | 5 | 3 (60.0) | 1 (20.0) | 0 (0.0) | 0 (0.0) | 1 (20.0) | 0 (0.0) |  |
| Intellectual or autistic disability |  |  |  |  |  |  |  |  |
| Grade 1–3 | 220 | 98 (44.6) | 30 (13.6) | 0 (0.0) | 0 (0.0) | 21 (9.6) | 71 (32.3) |  |
| Grade 4–6 | – | – | – | – | – | – | – |  |
| Mental disability |  |  |  |  |  |  |  |  |
| Grade 1–3 | 248 | 117 (47.2) | 29 (11.7) | 0 (0.0) | 0 (0.0) | 22 (8.9) | 80 (32.3) |  |
| Grade 4–6 | – | – | – | – | – | – | – |  |
| Cardiac disability |  |  |  |  |  |  |  |  |
| Grade 1–3 | 114 | 62 (54.4) | 7 (6.1) | 0 (0.0) | 1 (0.9) | 3 (2.6) | 41 (36.0) |  |
| Grade 4–6 | 2 | 1 (50.0) | 0 (0.0) | 0 (0.0) | 0 (0.0) | 0 (0.0) | 1 (50.0) |  |
| Pulmonary disability |  |  |  |  |  |  |  |  |
| Grade 1–3 | 149 | 86 (57.7) | 5 (3.4) | 0 (0.0) | 0 (0.0) | 6 (4.0) | 52 (34.9) |  |
| Renal disability |  |  |  |  |  |  |  |  |
| Grade 1–3 | 346 | 200 (57.8) | 10 (2.9) | 0 (0.0) | 1 (0.3) | 13 (3.8) | 122 (35.3) |  |
| Grade 4–6 | 58 | 39 (67.2) | 1 (1.7) | 0 (0.0) | 1 (1.7) | 3 (5.2) | 14 (24.1) |  |
| Hepatic disability |  |  |  |  |  |  |  |  |
| Grade 1–3 | 12 | 4 (33.3) | 2 (16.7) | 0 (0.0) | 0 (0.0) | 0 (0.0) | 6 (50.0) |  |
| Grade 4–6 | 17 | 11 (64.7) | 1 (5.9) | 0 (0.0) | 1 (5.9) | 2 (11.8) | 2 (11.8) |  |
| Ostomy |  |  |  |  |  |  |  |  |
| Grade 1–3 | 6 | 1 (16.7) | 3 (50.0) | 0 (0.0) | 0 (0.0) | 1 (16.7) | 1 (16.7) |  |
| Grade 4–6 | 54 | 30 (55.6) | 0 (0.0) | 0 (0.0) | 0 (0.0) | 6 (11.1) | 18 (33.3) |  |
| Epilepsy disorder |  |  |  |  |  |  |  |  |
| Grade 1–3 | 15 | 9 (60.0) | 1 (6.7) | 0 (0.0) | 0 (0.0) | 0 (0.0) | 5 (33.3) |  |
| Grade 4–6 | 21 | 8 (38.1) | 4 (19.1) | 0 (0.0) | 0 (0.0) | 3 (14.3) | 6 (28.6) |  |

CT, chemotherapy; RT, radiotherapy; CRT, chemoradiotherapy.

**Supplementary Table 2. Characteristics of patients with resected gastric cancer.**

|  | **People without disabilities, n (%)** | **People with disability, n (%)** | **Disability severity, n (%)** | | **Disability type, n (%)** | | | | | | | | | |
| --- | --- | --- | --- | --- | --- | --- | --- | --- | --- | --- | --- | --- | --- | --- |
|  |  |  | **Grade 1–3** | **Grade 4–6** | **Physical** | | **Communi-cational** | | **Mental** | | **Internal organ** | | ^a^**Others** | |
| **All subjects** | 36,852 | 10,324 | 2,831 | 7,493 | 6,997 | | 2,624 | | 237 | | 439 | | 27 | |
| **Age, year** |  |  |  |  |  | |  | |  | |  | |  | |
| Mean (SD) | 64.6 (9.8) | 64.0 (9.8) | 63.1 (10.1) | 64.3 (9.7) | 64.0 (9.1) | | 66.5 (9.6) | | 55.5 (9.6) | | 62.2 (9.9) | | 56.2 (10.4) | |
| 19–40 | 463 (1.2) | 123 (1.2) | 51 (1.8) | 72 (1.0) | 81 (1.2) | | 23 (0.9) | | 11 (4.6) | | 6 (1.4) | | 2 (7.4) | |
| 41–65 | 15767 (42.8) | 4671 (45.2) | 1361 (48.1) | 3310 (44.2) | 3328 (47.5) | | 915 (34.9) | | 179 (75.5) | | 229 (52.2) | | 20 (74.1) | |
| 66–75 | 15395 (41.8) | 4216 (40.9) | 1095 (38.7) | 3121 (41.7) | 2839 (40.6) | | 1165 (44.4) | | 45 (19.0) | | 162 (36.9) | | 5 (18.5) | |
| >75 | 5227 (14.2) | 1314 (12.7) | 324 (11.4) | 990 (13.2) | 749 (10.7) | | 521 (19.8) | | 2 (0.9) | | 42 (9.6) | | 0 (0.0) | |
| **Sex** |  |  |  |  |  | |  | |  | |  | |  | |
| Male | 27623 (75.0) | 7724 (74.8) | 2242 (79.2) | 5482 (73.2) | 5144 (73.5) | | 2045 (77.9) | | 157 (66.2) | | 356 (81.1) | | 22 (81.5) | |
| Female | 9229 (25.0) | 2600 (25.2) | 589 (20.8) | 2011 (26.8) | 1853 (26.5) | | 579 (22.1) | | 80 (33.8) | | 83 (18.9) | | 5 (18.5) | |
| **CCI** |  |  |  |  |  | |  | |  | |  | |  | |
| Mean (SD) | 1.1 (1.5) | 1.5 (1.9) | 1.8 (2.1) | 1.4 (1.8) | 1.9 (2.0) | | 1.4 (1.8) | | 1.0 (1.6) | | 3.2 (2.5) | | 1.5 (2.0) | |
| 0 | 18824 (51.1) | 4303 (41.7) | 1112 (39.3) | 3191 (42.6) | 2896 (41.4) | (41.39) | | 1178 (44.9) | | 136 (57.4) | | 83 (18.9) | | 10 (37.1) |
| 1 | 7801 (21.2) | 2091 (20.3) | 507 (17.9) | 1584 (21.1) | 1433 (20.5) | (20.48) | | 550 (21.0) | | 48 (20.2) | | 51 (11.6) | | 9 (33.3) |
| 2 | 4513 (12.2) | 1438 (13.9) | 365 (12.9) | 1073 (14.3) | 1002 (14.3) | (14.32) | | 348 (13.2) | | 23 (9.7) | | 62 (14.1) | | 3 (11.1) |
| ≥ 3 | 5714 (15.5) | 2492 (24.1) | 847 (29.9) | 1645 (22.0) | 1666 (23.8) | (23.81) | | 548 (20.9) | | 30 (12.7) | | 243 (55.4) | | 5 (18.5) |
| **Comorbidity** |  |  |  |  |  | |  | |  | |  | |  | |
| Diabetes Mellitus | 5936 (16.1) | 2034 (19.7) | 599 (21.2) | 1435 (19.2) | 1334 (19.1) | | 520 (19.8) | | 25 (10.6) | | 151 (34.4) | | 4 (14.8) | |
| Hypertension | 14241 (38.6) | 4580 (44.4) | 1336 (47.2) | 3244 (43.3) | 3152 (45.1) | | 1101 (42.0) | | 45 (19.0) | | 275 (62.6) | | 7 (25.9) | |
| CHD | 4127 (11.2) | 1490 (14.4) | 485 (17.1) | 1005 (13.4) | 969 (13.9) | | 349 (13.3) | | 17 (7.2) | | 154 (35.1) | | 1 (3.7) | |
| Stroke | 1757 (4.8) | 1049 (10.2) | 419 (14.8) | 630 (8.4) | 787 (11.3) | | 212 (8.1) | | 12 (5.1) | | 35 (8.0) | | 3 (11.1) | |
| COPD | 4207 (11.4) | 1503 (14.6) | 469 (16.6) | 1034 (13.8) | 976 (14.0) | | 380 (14.5) | | 27 (11.4) | | 115 (26.2) | | 5 (18.5) | |
| **Income** |  |  |  |  |  | |  | |  | |  | |  | |
| Medicare | 1219 (3.3) | 1093 (10.6) | 572 (20.2) | 521 (7.0) | 640 (9.2) | | 236 (9.0) | | 143 (60.3) | | 63 (14.4) | | 11 (40.7) | |
| Lowest quartile | 8165 (22.2) | 2488 (24.1) | 604 (21.3) | 1884 (25.1) | 1711 (24.4) | | 643 (24.5) | | 30 (12.7) | | 100 (22.8) | | 4 (14.8) | |
| Second quartile | 7454 (20.2) | 2039 (19.8) | 492 (17.4) | 1547 (20.6) | 1456 (20.8) | | 475 (18.1) | | 19 (8.0) | | 86 (19.6) | | 3 (11.1) | |
| Third quartile | 9089 (24.7) | 2302 (22.3) | 581 (20.5) | 1721 (23.0) | 1589 (22.7) | | 593 (22.6) | | 23 (9.7) | | 94 (21.3) | | 3 (11.1) | |
| Highest quartile | 10925 (29.6) | 2402 (23.2) | 582 (20.6) | 1820 (24.3) | 1601 (22.9) | | 677 (25.8) | | 22 (9.3) | | 96 (21.9) | | 6 (22.3) | |
| **Residence** |  |  |  |  |  | |  | |  | |  | |  | |
| Metropolitan | 21749 (59.0) | 5578 (54.0) | 1529 (54.0) | 4049 (54.0) | 3759 (53.7) | | 1427 (54.4) | | 116 (49.0) | | 265 (60.4) | | 11 (40.7) | |
| City | 10082 (27.4) | 3107 (30.1) | 890 (31.4) | 2217 (29.6) | 2131 (30.5) | | 762 (19.0) | | 82 (34.6) | | 122 (27.8) | | 10 (37.1) | |
| Rural | 5021 (13.6) | 1639 (15.9) | 412 (14.6) | 1227 (16.4) | 1107 (15.8) | | 435 (16.6) | | 39 (16.4) | | 52 (11.9) | | 6 (22.2) | |
| **SEER** |  |  |  |  |  | |  | |  | |  | |  | |
| Localized | 25797 (70.0) | 7280 (70.5) | 1951 (68.9) | 5329 (71.1) | 4958 (70.9) | | 1835 (69.9) | | 152 (64.1) | | 315 (71.8) | | 20 (74.1) | |
| Locoregional | 11055 (30.0) | 3044 (29.5) | 880 (31.1) | 2164 (28.9) | 2039 (29.1) | | 789 (30.1) | | 85 (35.9) | | 124 (28.2) | | 7 (25.9) | |
| **Treatment** |  |  |  |  |  | |  | |  | |  | |  | |
| Surgery alone | 31746 (86.1) | 8984 (87.0) | 2452 (86.6) | 6532 (87.2) | 6064 (86.7) | | 2294 (87.4) | | 191 (80.6) | | 412 (93.9) | | 23 (85.2) | |
| Surgery + CT | 4775 (13.0) | 1269 (12.3) | 361 (12.8) | 908 (12.1) | 877 (12.5) | | 318 (12.1) | | 46 (19.4) | | 24 (5.5) | | 4 (14.8) | |
| Surgery + RT | 41 (0.1) | 11 (0.1) | 5 (0.2) | 6 (0.1) | 10 (0.1) | | 1 (0.1) | | 0 (0.0) | | 0 (0.0) | | 0 (0.0) | |
| Surgery + CRT | 290 (0.8) | 60 (0.6) | 13 (0.5) | 47 (0.6) | 46 (0.7) | | 11 (0.4) | | 0 (0.0) | | 3 (0.6) | | 0 (0.0) | |
| **Screening subjects** | 28,864 | 7,435 | 1,692 | 5,743 | 5,210 | | 1,912 | | 88 | | 212 | | 13 | |
| **Smoking** |  |  |  |  |  | |  | |  | |  | |  | |
| No | 14068 (48.6) | 3798 (51.1) | 838 (49.5) | 2960 (51.5) | 2666 (51.2) | | 967 (50.6) | | 53 (60.2) | | 106 (50.0) | | 6 (46.1) | |
| Past | 6737 (23.3) | 1664 (22.4) | 429 (25.4) | 1235 (21.5) | 1125 (21.6) | | 459 (24.0) | | 7 (8.0) | | 70 (33.0) | | 3 (23.1) | |
| Current | 8059 (27.9) | 1973 (26.5) | 425 (25.1) | 1548 (27.0) | 1419 (27.2) | | 486 (25.4) | | 28 (31.8) | | 36 (17.0) | | 4 (30.8) | |
| **BMI, kg/m^2^** |  |  |  |  |  | |  | |  | |  | |  | |
| ＜18.5 | 958 (3.3) | 245 (3.3) | 62 (3.7) | 183 (3.2) | 148 (2.8) | | 85 (4.5) | | 4 (4.5) | | 8 (3.8) | | 0 (0.0) | |
| 18.5–23 | 10408 (36.1) | 2521 (33.9) | 642 (37.9) | 1879 (32.7) | 1676 (32.2) | | 725 (37.9) | | 29 (32.9) | | 88 (41.5) | | 3 (23.0) | |
| 23–25 | 7704 (26.7) | 1907 (25.7) | 436 (25.8) | 1471 (25.6) | 1322 (25.4) | | 496 (25.9) | | 24 (27.3) | | 60 (28.3) | | 5 (38.5) | |
| 25–30 | 9022 (31.2) | 2486 (33.4) | 504 (29.8) | 1982 (34.5) | 1846 (35.4) | | 558 (29.2) | | 26 (29.6) | | 51 (24.1) | | 5 (38.5) | |
| >30 | 772 (2.7) | 276 (3.7) | 48 (2.8) | 228 (4.0) | 218 (4.2) | | 48 (2.5) | | 5 (5.7) | | 5 (2.3) | | 0 (0.0) | |

^a^Others: facial disfigurement and epilepsy disorder.

SD, standard deviation; CCI, Charlson comorbidity index; CHD, coronary heart disease; COPD, chronic obstructive pulmonary disease; SEER, Surveillance, Epidemiology, and End Results; CT, chemotherapy; RT, radiotherapy; CRT, chemoradiotherapy; BMI, body mass index.
